# Validation of Brunei’s Malay EQ-5D Questionnaire in Patients with Type 2 Diabetes

**DOI:** 10.1371/journal.pone.0165555

**Published:** 2016-11-11

**Authors:** David Koh, Awg Muhammad Khairulamin bin Abdullah, Pei Wang, Naing Lin, Nan Luo

**Affiliations:** 1 PAPRSB Institute of Health Sciences, Universiti Brunei Darussalam, Bandar Seri Begawan, Brunei; 2 SSH School of Public Health, National University of Singapore, Singapore, Singapore; Soochow University Medical College, CHINA

## Abstract

**Background:**

The Malay spoken in Brunei a South East Asian country where Malay is the national language is distinctive and different from Malay spoken in Malaysia, Singapore and Indonesia. This study aimed to develop a Brunei Malay version of the 5-level EQ-5D questionnaire (EQ-5D-5L) and to assess its psychometric properties among patients with type 2 diabetes mellitus (T2DM).

**Methods:**

The Brunei Malay EQ-5D-5L was developed by culturally adapting two existing Malay versions. A total of 154 Bruneians with T2DM completed the questionnaire in two different points of time with one week apart. Known-groups validity of the utility-based EQ-5D-5L index and visual analogue scale (EQ-VAS) was evaluated by comparing subgroups of patients known to differ in health status. Test-retest reliability was assessed using the intraclass correlation coefficient (ICC) or Cohen’s kappa.

**Results:**

As hypothesized, patients known to have ‘better’ health had higher EQ-5D-5L index scores than those having ‘worse’ health in all 7 known-groups comparisons. The hypothesized difference in the EQ-VAS scores was observed in only 4 of the 7 known-groups comparisons. Kappa values ranged from 0.206 to 0.446 for the EQ-5D-5L items; the ICC value for the EQ-5D-5L index and EQ-VAS was 0.626 and 0.521, respectively.

**Conclusions:**

The utility-based EQ-5D-5L index appears to be valid and reliable for measuring the health of Brunei patients with T2DM. The validity of the EQ-VAS in Brunei requires further investigation.

## Introduction

Type 2 diabetes mellitus (T2DM) is a common disease in Brunei, a South East Asian country. It affects 11% of the Bruneian population [1] and is the third leading cause of mortality after cancer and coronary heart diseases [2]. Given its popularity and association with impaired health-related quality of life (HRQoL) [3], it is important to assess HRQoL of T2DM patients in Brunei using an instrument which has been cross-culturally adapted for use in local context.

The 5-level EQ-5D questionnaire (EQ-5D-5L) is a new version of the EQ-5D [4] which has been the most widely utilised generic HRQoL instrument in diabetes patients [5]. The EQ-5D-5L has exhibited higher sensitivity than the original version of EQ-5D in T2DM patients [6]. It provides a multidimensional profile, a single utility-based index, and an overall self-assessment of health. At present, the EQ-5D-5L has been available in over 100 languages, including Malay for use in the Malay-speaking populations of Singapore and Malaysia but not of Brunei Darussalam [7].

Malay spoken in Brunei is distinctive and different from Malay spoken in Malaysia, Singapore and Indonesia. It has a lexical difference of 18–20% with Standard Malay spoken in neighbouring Malaysia and Singapore [8]. We aimed to develop a Malay version of the EQ-5D-5L for Brunei and to assess its psychometric properties in patients with type 2 diabetes mellitus (T2DM).

## Methods

This study used the design of cross-sectional survey, adhered to the tenets of the Helsinki Declaration, and was approved by the Medical and Health Research Ethics Committee, Ministry of Health, Brunei.

### Sampling

Purposive sampling was employed to obtain a heterogeneous sample of T2DM patients. Patients were recruited from the endocrine wards Raja Isteri Pengiran Anak Saleha (RIPAS) Hospital, the specialist diabetic outpatient clinic in RIPAS Hospital, Sengkurong Primary Health Centre and Rimba Primary Health. RIPAS is the national hospital of Brunei, which has more than 600 beds and offers a wide range of health services to the indigenous population.

The inclusion criteria were a diagnosis of T2DM, age above 20 years, and ability to read and communicate in Brunei spoken Malay. The exclusion criteria included inability to answer questions due to debilitating health conditions and the main reason for admission or visit not being T2DM or related health problems.

### Data collection procedure

After informed consent was obtained, all patients were asked to complete the EQ-5D-5L and questions assessing their demographic and diabetic status in the ward or clinic where they were recruited. Assistance from a trained research assistant was provided whenever necessary. After the questionnaire survey, a saliva sample was taken from each patient and subsequently analysed using a commercial test kit for the concentration of cortisol, a biomarker for the level of stress [9].

All patients were invited to complete the EQ-5D-5L again one week after the baseline survey via telephone, post, email, test messaging or Whatsapp communication, depending on patients’ own preferences. Up to three reminders were sent to non-respondents.

### Instrument

The EQ-5D-5L consists of five Likert-type items and a visual analogue scale (aka EQ-VAS). Each item asks respondents to rate their HRQol in a different dimension (mobility, self-care, usual activities, pain/discomfort, or anxiety/depression) as having no problems, slight problems, moderate problems, severe problems, or extreme problems. Responses to the 5 items can be used to calculate a single index score anchored by 0 (dead) and 1 (full health). This score indicates the utility or value of the respondent’s health from the perspective of the general public [10]. The EQ-VAS is a 20cm, vertical scale numbered from 0 to 100 for respondents to assess their overall health on the day of the survey, where 0 and 100 are labelled with ‘the worst imaginable health state’ and ‘the best imaginable health state’, respectively.

The Brunei Malay version of the EQ-5D-5L questionnaire was developed using the cultural adaptation procedure recommended by the EuroQol Group. It involved firstly proposing the wording for a Brunei Malay EQ-5D-5L based on the Singapore and Malaysia Malay versions of the questionnaire [11] by one of the investigators of this study and researchers from the Malay Language and Linguistics Department of University Brunei Darussalam who had experience in questionnaire translation. Secondly, an independent reviewer with a health background checked the Brunei Malay version for the appropriateness for use in the clinical setting. Thirdly, a pilot study was conducted with 16 Brunei Malay-speaking persons whose professions were not health related. The feedback collected from the pilot study was used to finetune the wording of the questionnaire. Lastly, the entire process was documented in a report which was subsequently reviewed and approved by a translation consultant appointed by the EuroQol Group. This was to ensure that the Brunei Malay version is semantically equivalent to the original English version of the questionnaire.

### Data analysis

The study sample was described in terms of frequency and proportion for categorical variables and mean and standard deviation for continuous variables. The EQ-5D-5L health index score was calculated by first mapping the EQ-5D-5L health profiles to EQ-5D-3L profiles using an algorithm developed by van Hout et al [12] and then applying the EQ-5D-3L value set of Singapore [13], which is a neighbouring country of Brunei.

Known-groups validity of the EQ-5D-5L health index and EQ-VAS was evaluated by comparing subgroups of patients known to differ in health status. The known groups were defined according to smoking status (yes versus no) [14], body mass index (obese versus non-obese) [15], use of insulin (yes versus no) [16], duration of diabetes (<10 years versus 10 or more years) [17], presence of diabetes-related complications (yes versus no) [17], presence of comorbidity (yes versus no) [17], and level of cortisol concentration (<0.10 versus ≥0.10), which is a predictor of depression [9]. We hypothesized that both scores would be lower in patients known to have 'worse' health than those who had 'better' health. Taking insulin usage as an example, we hypothesized that patient who used insulin (the group in ‘worse’ health) would have lower health index and EQ-VAS scores than those who did not use insulin (the group in ‘better’ health’). Linear regression models were used to examine the differences in EQ-5D health index and EQ-VAS scores between each pair of known-groups with the adjustment of age, gender, and race.

Test-retest reliability was assessed for the EQ-5D-5L health index and EQ-VAS using intraclass correlation coefficient (ICC) and for the five EQ-5D-5L items using Cohen’s kappa. For both the two measures [18, 19], values being 0–0.20, 0.21–0.40, 0.41–0.60, 0.61–0.80, and 0.81–1 indicate slight agreement, fair agreement, moderate agreement, substantial agreement, and almost perfect agreement, respectively. All statistical tests were two-sided with the significance level set at 0.01.

All analyses were conducted using SAS (version 9.3).

## Results

One hundred and fifty-four patients completed the baseline survey, and among those, 116 also completed the follow-up survey during the period of February to June 2014.

The patients’ socio-demographic and clinical characteristics and responses to the EQ-5D-5L items are summarized in Tables 1 and 2. The majority of patients were middle aged (45–64 years) [20], female, and ethnic Malay. A significant proportion of the patients reported having at least one complication and no less than two comorbidities. Most patients reported “no problems” with each of the five EQ-5D-5L items.

**Table 1:** Patients’ characteristics for the baseline (*n* = 154) and follow-up (*n* = 116) surveys.

Variable	Baseline *n* (%)	Follow-up *n* (%)
**Age, years**	52.3 (12.0)^a^	51.8 (12.0)^a^
<45	43 (27.9)	34 (29.3)
45–64	88 (57.1)	66 (56.9)
65+	23 (14.9)	16 (13.8)
**Gender**		
Male	50 (32.5)	36 (31.0)
Female	104 (67.5)	80 (69.0)
**Race**		
Malay	139 (90.3)	106 (91.4)
Others	15 (9.7)	10 (8.6)
**Marital status**		
Married	135 (87.7)	100 (86.2)
Not married	19 (12.3)	16 (13.8)
**Occupation**		
Unemployed	8 (5.2)	5 (4.3)
Retired	38 (24.7)	25 (21.6)
Employed	79 (51.3)	65 (56.0)
Housewife	25 (16.2)	18 (15.5)
Others	4 (2.6)	3 (2.6)
**Income (BND**^**b**^**)**		
<$1000	16 (10.4)	15 (12.9)
$1000-$1999	25 (16.2)	18 (15.5)
$2000-$3999	23 (14.9)	17 (14.7)
>$4000	17 (11.0)	13 (11.2)
**Education**		
No formal qualification	25 (16.2)	17 (14.7)
Primary (PSR)	37 (24.0)	25 (21.6)
Secondary (‘O’/’N’ or ‘A’ levels)	62 (40.3)	51 (44.0)
Tertiary	30 (19.5)	23 (19.8)
**Study sites**		
Primary Health Centre	23 (14.9)	13 (11.7)
Diabetic Clinic	111 (72.1)	85 (76.6)
Endocrine Ward	20 (13.0)	18 (16.2)
**Number of complications**		
0	50 (32.5)	38 (33.0)
1	52 (33.8)	39 (33.9)
2	32 (20.8)	22 (19.1)
3	20 (13.0)	16 (13.9)
**Number of comorbidity**		
0–1	51 (33.1)	34 (29.6)
2–3	91 (59.0)	71 (61.7)
4–7	11 (7.1)	9 (7.8)
**BMI**	30.20 (7.40)^a^	30.30 (7.30)^a^
**Duration of DM, year**	11.20 (7.80)^a^	10.90 (7.60)^a^
**Cortisol, ug/dL**	0.16 (0.22)^a^	0.15 (0.20)^a^
Full health*	82 (53.3)	72 (62.1)

^a^ Mean (SD)

^b^ 1 BND = 0.71 USD

*Full health is defined as no problems on all the EQ-5D-5L domains.

**Table 2:** Self-reported EQ-5D-5L problems in the baseline and follow-up surveys.

	Baseline survey (*n* = 154)		Follow-up survey (*n* = 116)	
	*n*	(%)	*n*	(%)
**Mobility**				
No	108	(70.1)	96	(82.8)
Slight	32	(20.8)	15	(12.9)
Moderate	11	(7.1)	4	(3.4)
Severe	2	(1.3)	1	(0.9)
Unable	1	(0.6)	0	(0.0)
**Self-care**				
No	143	(92.9)	110	(94.8)
Slight	6	(3.9)	4	(3.4)
Moderate	5	(3.2)	2	(1.7)
Severe	0	(0.0)	0	(0.0)
Unable	0	(0.0)	0	(0.0)
**Usual activities**				
No	134	(87.0)	101	(87.1)
Slight	14	(9.1)	11	(9.5)
Moderate	2	(1.3)	3	(2.6)
Severe	3	(1.9)	0	(0.0)
Unable	1	(0.6)	1	(0.9)
**Pain-discomfort**				
No	104	(67.5)	82	(70.7)
Slight	40	(26.0)	26	(22.4)
Moderate	9	(5.8)	7	(6.0)
Severe	1	(0.6)	1	(0.9)
Extreme	0	(0.0)	0	(0.0)
**Anxiety/depression**				
Not	124	(80.5)	93	(80.2)
Slightly	25	(16.2)	17	(14.7)
Moderately	4	(2.6)	4	(3.4)
Severely	1	(0.6)	1	(0.9)
Extremely	0	(0.0)	1	(0.9)

### Validity

The validity analyses were based on all the 154 patients. In all known-groups comparisons, the ‘better’ group was found to have a higher mean EQ-5D-5L index score than the ‘worse’ group (Table 3). Statistical significance was reached in 4 of the 7 comparisons (i.e. smoking status, years of DM, complications, and comorbidities). Nevertheless, the mean EQ-VAS score was higher for the ‘better’ group than for the ‘worse’ group in only 4 of the 7 comparisons (i.e. insulin, complications, comorbidities, and cortisol concentration), and none of the comparisons showed a statistical significant difference (Table 3).

**Table 3:** Association of health and clinical variables with EQ-5D-5L health index and EQ-VAS (*n* = 154).

Variable	*n* (%)	Index score Mean (SD)	EQ-VAS score Mean (SD)
**Smoking status**			
Never	121 (78.6)	0.87 (0.19) ^a^	79.68 (16.51)
Current or ex-smoker	33 (21.4)	0.76 (0.30) ^a^	80.88 (12.53)
**BMI**			
< 30 (non-obese)	90 (58.4)	0.87 (0.20)	78.41 (16.84)
> = 30 (obese)	64 (41.6)	0.82 (0.25)	82.08 (13.81)
**Years of DM**			
< 10 years	72 (46.8)	0.90 (0.14)^b^	79.21 (18.29)
> = 10 years	81 (52.6)	0.81 (0.26)^b^	80.33 (13.02)
**Insulin**			
No	81 (52.6)	0.87 (0.21)	81.23 (15.49)
Yes	73 (47.4)	0.82 (0.23)	78.49 (15.93)
**Complications**			
No	50 (32.5)	0.91 (0.16)^c^	81.50 (14.26)
Yes	104 (67.5)	0.81 (0.24)^c^	79.18 (16.37)
**Comorbidities**			
< 3 comorbidities	112 (72.7)	0.89 (0.17)^d^	80.11 (16.16)
> = 3 comorbidities	41 (26.6)	0.72 (0.29)^d^	79.71 (14.72)
**Cortisol concentration (ug/dL)**			
<0.10	63 (40.9)	0.88 (0.20)	80.68 (12.39)
> = 0.10	87 (56.5)	0.82 (0.23)	79.33 (18.06)

^a,b,c,d^ *p*< 0.01 in multivariate linear regression analysis adjusted for age, gender, and race.

### Test-retest reliability

The reliability analyses were based on the 116 patients who completed both the baseline and follow-up survey. Cohen’s kappa value was 0.340 for the mobility item, 0.440 for the self-care item, 0.446 for the usual activities item, 0.206 for the pain/discomfort item, and 0.367 for the anxiety/depression item. The ICC value for the EQ-5D-5L health index and EQ-VAS was 0.626 and 0.521, respectively.

## Discussion

Any health questionnaire must be assessed when it is introduced to a new socio-cultural setting for which it is not originally developed. Therefore, we developed the EQ-5D-5L questionnaire into Brunei Malay and tested it in this Southeast Asian country. To the best of our knowledge, no instruments for outcomes research were validated for use in Brunei.

Known-groups validity is a form of construct validity which is assessed when there is no a gold standard measure available for validating new HRQoL instruments [21]. Our a-priori hypotheses for the pre-defined known groups were all fulfilled for the EQ-5D-5L health index, indicating construct validity of this measure. This result suggests that the validity of Singapore Malay version of the EQ-5D-5L [22] was not lost after it was adapted for use in a different Malay-speaking population. It should be noted that statistical significance was not used as prerequisite for the fulfilment of the hypotheses. Statistical significance is not the primary consideration in construct validity testing; instead, it is more important to make and test as many hypotheses as possible with the available data [23], which may lead to insignificant results for some tests due to insufficient statistical power. On the other hand, the validity of the EQ-VAS cannot be confirmed as the hypotheses were not fulfilled in some known groups. It was not very surprising as similar results were reported in Singapore [24, 25]. Hence, the validity of the EQ-VAS in Brunei warrants further investigation.

Based on recommended interpretation guide for Cohen’s kappa and ICC values [22], the test-retest reliability of the EQ-5D-5L items, index, and EQ-VAS are acceptable. However, the degree of reliability exhibited in our study was lower than that reported for Chinese (kappa value: 0.73 to 0.98) and Korean (kappa value: 0.41 to 0.69) versions of the EQ-5D-5L [26, 27]. The inferior test-retest reliability results in our study could be due to the different administration modes and locations of the baseline and follow-up surveys. It is possible that the health status of some patients changed from baseline to follow-up surveys. Theoretically, such patients should be excluded from the test-retest reliability assessment; however, we could not identify them. Hence, the actual reliability of the Brunei Malay EQ-5D-5L questionnaire could be underestimated. It should be noted that slightly lower reliability would not negatively affect the validity of the measures but would affect its sensitivity or discriminatory power [28]. This means that a less reliable measure requires a larger sample size than a more reliable measure to achieve certain statistical power when they are used to detect a difference between two populations.

The limitations of the study included the use of foreign algorithms for calculating the EQ-5D-5L index score and that the responsiveness of the EQ-5D-5L measures was not assessed. Also, the moderate sample size may have limited statistical power to detect differences for known-groups comparisons.

## Conclusions

In conclusion, the utility-based health index generated from the Brunei Malay EQ-5D-5L questionnaire has acceptable validity and reliability in measuring the health status of type 2 diabetes patients, supporting its use in this population. However, the validity of the EQ-VAS in Brunei type 2 diabetes patients requires further investigation in future studies.
